# Genetic Etiology Study of Ten Chinese Families with Nonsyndromic Hearing Loss

**DOI:** 10.1155/2018/4920980

**Published:** 2018-07-05

**Authors:** Songqun Hu, Feifei Sun, Jie Zhang, Yan Tang, Jinhong Qiu, Zhixia Wang, Luping Zhang

**Affiliations:** Department of Otolaryngology, Affiliated Hospital of Nantong University, Nantong, Jiangsu Province, China

## Abstract

Nonsyndromic hearing loss has been shown to have high genetic heterogeneity. In this report, we aimed to disclose the genetic causes of the subjects from the ten Chinese deaf families who did not have pathogenic common genes/mutation. Next-generation sequencing (NGS) of 142 known deafness genes was performed in the probands of ten families followed by cosegregation analysis of all family members. We identified novel pathogenic variants in six families including p.D1806E/p.R1588W, p.R964W/p.R1588W, and p.G17C/p.G1449D in *CDH23*; p.T584M/p.D1939N in *LOXHD1*; p.P1225L in *MYO7A*; and p.K612X in *EYA4*. Sanger sequencing confirmed that these mutations segregated with the hearing loss of each family. In four families, no pathogenic variants were identified. Our study provided better understanding of the mutation spectrum of hearing loss in the Chinese population.

## 1. Introduction

Hearing loss (HL) is the most common sensory disorder in humans, affecting one in every 500 newborns. Genetic causes account for at least 50% to 60% of childhood HL [1]. An accurate genetic diagnosis provides many immediate and long-term advantages to patients and their families [2]. Hereditary HL, however, is a highly heterogenous disorder. To date, over 90 genes have been identified as responsible for nonsyndromic sensorineural hearing loss (NSHL, http://hereditaryhearingloss.org/). In China, mutations in many genes have been found associated with hereditary HL, and the mutation spectrums were broad and diverse [3].Genetic heterogeneity and the small size of families with hereditary HL have hindered the unravelling of the genetic causes. Recently, the advent of targeted DNA capturing and next-generation sequencing (NGS) may make it possible to analyze most, if not all, deafness genes, as opposed to screening of each individual gene by conventional Sanger sequencing [2–4]. Using this strategy, we analyzed ten Chinese families with hereditary hearing loss and disclose their genetic causes.

## 2. Methods

### 2.1. Family Description and Clinical Evaluations

**Figure 1:**
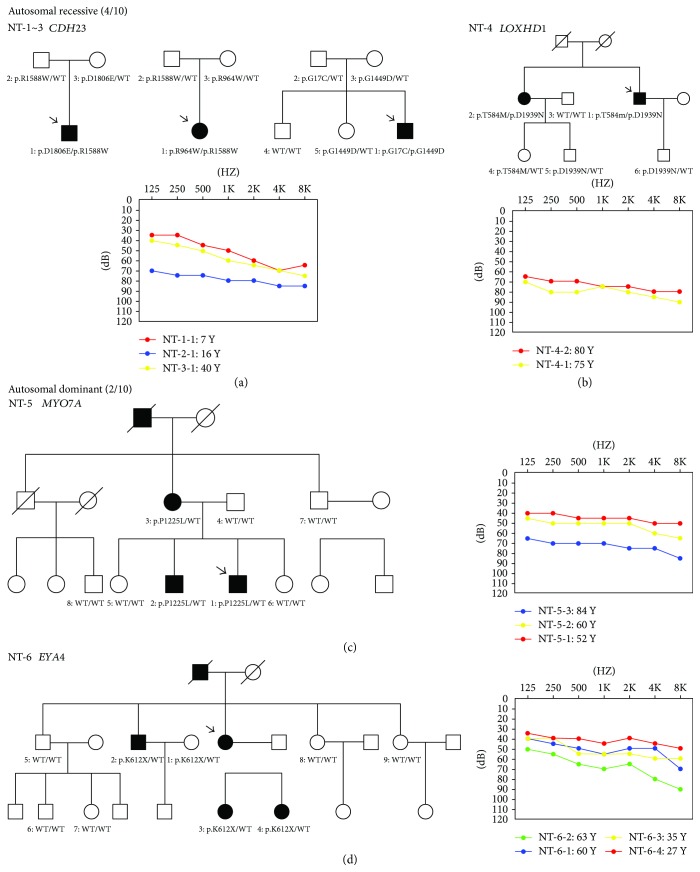
Pedigrees, genetic findings, and audiograms for NT-1~3 (a), NT-4 (b), NT-5 (c), and NT-6 (d). The arrow shows the probands in each family.

**Figure 2:**
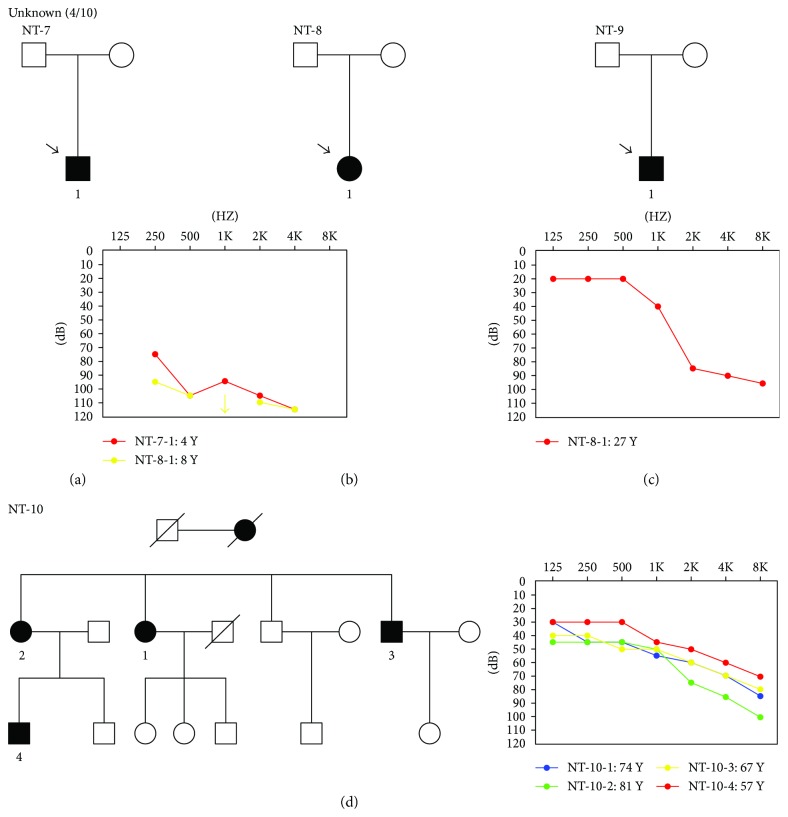
Pedigrees and audiometric features of family NT-7 (a), NT-8 (b), NT-9 (c), and NT-10 (d).

The ten families (NT-1~10) were recruited from the Department of Otolaryngology, Affiliated Hospital of Nantong University, Nantong, China. The pedigrees of those hearing loss-affected families of Han origin are shown in Figures 1 and 2. All patients had bilateral, symmetrical sensorineural hearing loss (SNHL). They received comprehensive medical history inquiry and thorough exams of auditory sense, vestibular function, and ophthalmic function, to rule out any possible environmental factors or syndromic hearing loss. All affected individuals were evaluated through detailed audiological evaluations including otoscopy, pure-tone audiometry, auditory brainstem response (ABR), distortion product otoacoustic emissions (DPOAEs), and auditory steady-state response (ASSR) test in subjects with very young age. The hearing loss level was classified as described previously [3]. Computed tomography (CT) scans were performed in the ten probands. This study was approved by the Ethics Committee of the Affiliated Hospital of Nantong University. All subjects gave written informed consent to participate in this study from October 1, 2014, to December 31, 2017.

### 2.2. Targeted Massively Parallel Sequencing

Genomic DNAs from all the family members available were extracted from whole peripheral blood leukocytes using a DNA extraction kit (Tiangen Biotech, China). Mutation screening of *GJB2*, *SLC26A4*, and the mitochondrial 12S rRNA was conducted first in ten probands using polymerase chain reaction (PCR) amplification, and exons were sequenced directly. All 10 probands of the deaf families were subjected to a gene panel containing 142 deafness-related genes by NGS (Supplementary 1). Targeted gene capturing, data processing, bioinformatic analysis, and filtering against multiple databases for SNPs were performed as reported in detail previously [3]. Potential causative variants detected by targeted massively parallel sequencing were confirmed by Sanger sequencing in each proband. Cosegregation analysis was also performed for multiplex probands in all family individuals if available.

## 3. Results

### 3.1. Clinical Manifestations

All the patients in the ten Chinese families showed bilateral, symmetrical, nonsyndromic, sensorineural hearing impairment. The age of the affected family members ranged from 4 to 84 years. The age at onset of hearing loss in these patients ranged from congenital to 42 years old. The hearing loss was symmetric while there was a wide range of different degrees of HL including moderate, severe, and profound. The patients, who carried compound heterozygous mutations in *CDH23* (NT-1-1, NT-2-1, and NT-3-1) and *MYO7A* (NT-5-1), two genes associated with both nonsyndromic deafness and Usher syndrome type 1, did not show any degenerative symptoms of retinitis pigmentosa. No inner ear malformation was observed by CT scanning.

### 3.2. Mutation Analysis

The results of screening of the three common genes in the probands of the 10 families were all negative. Targeted NGS of 142 hearing loss-related genes was carried out in the ten probands. Briefly, to detect possible causative mutations, variants meeting the criteria were filtered out as previously described [3]. Possible causative variants were summarized in Supplementary 2. The novel compound heterozygous variants were verified in four recessive families, including p.R1588W/p.D1806E of *CDH23* in NT-1-1, p.R964W/p.R1588W of *CDH23* in NT-2-1, p.G17C/p.G1449D of *CDH23* in NT-3-1, and p.T584M/p.D1939N of *LOXHD1* in NT-4-1. In addition, we identified two heterozygous candidate mutations p.P1225L in *MYO7A* and p.K612X in *EYA4* in two dominant probands NT-5-1 and NT-6-2, respectively. Sanger sequencing of available family members revealed that these mutations were present in all affected family individuals but not in the normal individuals (Supplementary 3).

Among the nine candidate mutations, p.R1588W, p.D1806E, and p.G1449D in *CDH23* have been reported to be pathogenic in previous reports [4–6]. As for the other six novel variants, p.K612X in *EYA4* was predicted to result in EYA4 eyaHR deletion, while p.G17C and p.R964W in *CDH23*, p.T584M and p.D1939N in *LOXHD1*, and p.P1225L in *MYO7*A were unanimously evaluated to be possibly damaging or disease-causing by more than two of the bioinformatic programs, such as the Mutation Taster, SIFT, and PolyPhen2. Our data indicated that six variants in *CDH23*, *LOXHD1*, *MYO7A*, and *EYA4* were likely to be pathogenic rather than a polymorphism. (Table 1, Supplementary 4). No disease-causing variants were identified in families NT-7, NT-8, NT-9, and NT-10.

## 4. Discussion

In this study, biallelic mutations in *CDH23* were identified by targeted NGS in 3 of the 10 families. Mutations in *CDH23* are the pathogenic cause for both Usher syndrome 1D (USH1D) and autosomal recessive nonsyndromic hearing loss (DFNB12). Patients with DFNB12 usually carry *CDH23* missense mutations in any domain, whereas individuals with USH1D usually have nonsense, splice-site, and frameshift mutations [5–7]. To date, at least 80 pathogenic variants of the *CDH23* have been reported in familial or sporadic patients of USH1D and DFNB12 worldwide. Ethnic diversity of genetic variance has been reported in deafness gene *CDH23* [4-8]. However, few of these mutations were detected in the Chinese population [3]. In this study, three compound heterozygous mutations (p.R1588W/p.D1806E, p.G17C/p.G1449D, and p.R964W/p.R1588W) in *CDH23* were identified by targeted NGS in two patients with moderate HL and one with severe HL. At the time of this report, the patients NT-1-1, NT-2-1, and NT-3-1 who carried the compound heterozygous *CDH23* mutations were 7, 16, and 43 years, respectively. No visual problems and vestibular dysfunction were revealed in any of them. Nevertheless, especially for young probands NT-1-1 and NT-2-1, we cannot definitely rule out that they would develop retinopathy later in their life. In previous studies, audiological phenotypes of *CDH23* compound heterozygotes seemed to be highly variable [8, 9]. Similarly in our study, NT-1-1 and NT-2-1 manifested prelingual-onset SNHL, while NT-3-1 showed adult-onset progressive SNHL that was not noticeable until age 30. The two probands (NT-1-1, NT-2-1) both used a hearing aid with satisfactory effect. They have normal conversations and are enrolled in regular school. In addition, we identified a relatively high prevalence (3/10) of *CDH23* mutations in Chinese Han population, and our report of the two novel mutations expanded the *CDH23* mutation spectrum.

*LOXHD1* mutations have been highly rare, which are known to be the cause of DFNB77. Up to date, there have been only six studies [10]. In the present study, we identified c.1751C>T (p.T584M) and c.5815G>A (p.D1939N) as novel, possibly pathogenic *LOXHD1* mutations, which cosegregated with the disease. It was predicted as possibly pathogenic by PolyPhen2, SIFT, and Mutation Taster. This is the first reported *LOXHD1* mutation causing hearing loss in China. In family NT-4, two affected siblings (NT-4-1, male, 75 years old; NT-4-2, female, 80 years old) had experienced bilateral slowly progressive hearing loss with onset of 35–40 yrs. Additionally, they both reported troublesome tinnitus. Phenotype-wise, compared to the previous reports [10, 11], our cases had milder and progressive hearing impairment.

The *MYO7A* gene mutations have been reported as the cause of Usher syndrome type 1B (USH1B), a syndromic deafness combined with retinitis pigmentosa and vestibular abnormalities [12]. *MYO7A* is also associated with nonsyndromic hearing loss (DFNB2, DFNA11) [13–15]. More recently, only eight mutations in the *MYO7A* gene have been identified associated with DFNA11 [15]. Here, we identify a novel missense variants (c.3674C>T, p.P1225L) in a Chinese family with progressive SNHL affecting all frequencies. In this study, three affected subjects from family NT-5, who had the p.P1225L heterozygous mutation in *MYO7A*, were 84, 60, and 52 years at the test. The genetic defect segregating in this family shows autosomal dominant inheritance. The absence of vestibular and retinal defects and less severe hearing loss is consistent with the phenotype of a recently reported Chinese family [15]. Thus, we speculate this family has nonsyndromic hearing loss (DFNA11).

Another novel causative variant identified in the present study is p.K612X of *EYA4* segregating with dominant hearing loss in family NT-6. The *EYA4* gene is known to be responsible for both nonsyndromic deafness DFNA10 and syndromic deafness with dilated cardiomyopathy [16–18]. The novel p.K612X truncating mutation changed Lys612 to a stop codon, which was predicted to lead to a premature termination prior to the EYA homolog domains. It suggests that this nonsense mutation may inhibit normal development and maintenance of the organ of Corti and cause SNHL. The postlingual, progressive SNHL phenotype in family NT-6 is similar to what has been reported for four unrelated Chinese DFNA10 families [19–22]. Combining with previous studies, our study suggests that mutations in *EYA4* are not a relatively rare cause for autosomal dominant NSHL in the Chinese population, and our data provide more insights into the genotype-phenotype correlation between the truncating mutation of *EYA4* and the DFNA10 phenotype.

In this study, we were not able to obtain a genetic diagnosis for the other four families using the current NGS panel (Figure 2). Further studies including the whole-exome sequencing in the negative families could be useful to discover potential novel NSHL genes and to draw a complete molecular epidemiology picture.

## 5. Conclusions

In conclusion, we successfully identified novel and likely pathogenic mutations in six Chinese families by targeted NGS. Our result demonstrates that this new method is a highly effective molecular diagnostic tool for this heterogeneous disorder.

## Figures and Tables

**Table 1:** Mutations detected in six Chinese Han families.

Family ID	Gene	Mutation type	Nucleotide change (transcript version)	Amino acid change	Phylop score	Mutation taster	PROVEAN (score)	SIFT (score)	Allele frequency in controls	Novel or HGMD
Recessive										
NT-1	CDH23	Missense	c.4762C>T	p.R1588W	3.822	DC	Deleterious	Damaging	0/400	HGMD
			(NM_022124)				(−3.136)	−0.001		
	CDH23	Missense	c.5418C>G	p.D1806E	−1.832	DC	Neutral	Damaging	0/400	HGMD
			(NM_022124)				(−0.778)	−0.007		
NT-2	CDH23	Missense	c.2890C>T	p.R964W	0.855	DC	Deleterious	Damaging	0/400	Novel
			(NM_022124)				(−2.783)	−0.005		
	CDH23	Missense	c.4762C>T	p.R1588W	3.822	DC	Deleterious	Damaging	0/400	HGMD
			(NM_022124)				(−3.136)	−0.001		
NT-3	CDH23	Missense	c.49G>T	p.G17C	0.8	—	Deleterious	—	0/400	Novel
			NM_001171935				(−4.405)			
	CDH23	Missense	c.4346G>A	p.G1449D	5.967	*DC*	Deleterious	Tolerated	0/400	HGMD
			(NM_022124)				(−2.886)	−0.233		
NT-4	LOXHD1	Missense	c.1751C>T	p.T584 M	9.151	DC	Deleterious	Damaging	0/600	Novel
			(NM_144612)				(−4.6)	−0.001		
	LOXHD1	Missense	c.5815G>A	p.D1939N	7.672	DC	Deleterious	Damaging	0/600	Novel
			(NM_144612)				(−2.51)	−0.01		
Dominant										
NT-5	MYO7A	Missense	c.3674C>T	p.P1225L	5.846	DC	Deleterious	Damaging	0/600	Novel
			(NM_000260)				(−7.82)	−0.03		
NT-6	EYA4	nonsense	c.1834A>T	p.K612X	7.21	DC	—	—	0/600	Novel
			(NM_004100)							

## Data Availability

The data used to support the findings of this study are available from the corresponding author upon request.

## Supplementary Materials

Supplementary 1 Table S1: 142 genes targeted for the next-generation sequencing.

Supplementary 2 Table S2: all variants identified by targeted NGS.

Supplementary 3 Figure 1: validation of candidate mutations by PCR-Sanger sequencing.

Supplementary 4 Function analysis on candidate mutations.
