# Supplementary material for: Genetic Etiology Study of Ten Chinese Families with Nonsyndromic Hearing Loss
Source: Neural Plast. 2018 Jul 5;2018:4920980. doi: 10.1155/2018/4920980 (PMC6079373; doi:10.1155/2018/4920980)
Supplement: Supplementary 2 — Table S2: all variants identified by targeted NGS. [file 4920980.f2.doc]

**S2 Table**. All variants identified by targeted NGS

| **Proband** | **Gene** | **Ref_number** | **Nucletide**  **change** | **Amino acid**  **change** | **Genotype** | **Allele frequency**  **in controls** | **Cosegragation with the deafness** |
| --- | --- | --- | --- | --- | --- | --- | --- |
| **NT-1-1** | *CDH23* | NM_022124 | c.4762C>T | p.R1588W | Heterozygous | 0/400 | Yes |
|  |  |  | c.5418C>G | p.D1806E | Heterozygous | 0/400 | Yes |
|  | *COL4A3* | NM_000091 | c.3856G>A | p.G1286R | Heterozygous | 0/400 | No |
|  | *ILDR1* | NM_001199800 | c.802A>G | p.I268V | Heterozygous | 0/400 | No |
|  | *PTPRQ* | NM_001145026 | c.6115A>G | p.I2039V | Heterozygous | 0/400 | No |
|  | *DIAPH1* | NM_001079812 | c.1826_1827  insTCCTCCTCC | p.P609delinsPPPP | Heterozygous | 0/400 | No |
| **NT-2-1** | *CDH23* | NM_022124 | c.2890C>T | p.R964W | Heterozygous | 0/400 | Yes |
|  |  |  | c.4762C>T | p.R1588W | Heterozygous | 0/400 | Yes |
| **NT-3-1** | *CDH23* | NM_022124 | c.4346G>A | p.G1449D | Heterozygous | 0/400 | Yes |
|  |  | NM_001171935 | c.49G>T | p.G17C | Heterozygous | 0/400 | Yes |
|  | *DIAPH1* | NM_005219 | c.3614C>T | p.A1205V | Heterozygous | 0/400 | No |
| **NT-4-1** | *LOXHD1* | NM_144612 | c.5815G>A | p.D1939N | Heterozygous | 0/600 | Yes |
|  |  |  | c.1751C>T | p.T584M | Heterozygous | 0/600 | Yes |
|  | *COL2A1* | NM_001844 | c.415G>C | p.D139H | Heterozygous | 0/400 | No |
|  | *COL4A5* | NM_000495 | c.4847C>T | p.S1616F | Hemizygous | 0/400 | No |
| **NT-5-1** | *MYO7A* | NM_000260 | c.3674C>T | p.P1225L | Heterozygous | 0/600 | Yes |
|  | *NDRG1* | NM_006096 | c.634C>T | p.R212C | Heterozygous | 0/400 | No |
| **NT-6-1** | *EYA4* | NM_004100 | c.1834A>T | p.K612X | Heterozygous | 0/600 | Yes |
|  | *FLNA* | NM_001110556 | c.1327C>T | p.R443C | Heterozygous | 0/400 | No |
| **NT-7-1** | *CHD7* | NM_017780 | c.1565G>T | p.G522V | Heterozygous | 0/400 | No |
|  | *COL4A3* | NM_000091 | c.3627G>A | p.M1209I | Heterozygous | 0/400 | No |
|  | *COL4A5* | NM_000495 | c.2215C>G | p.P739A | Heterozygous | 0/400 | No |
|  | *KCNQ4* | NM_172163 | c.1656C>G | p.D552E | Heterozygous | 0/400 | No |
|  | *PROKR2* | NM_144773 | c.533G>C | p.W178S | Heterozygous | 0/400 | No |
|  | *PTPRQ* | NM_001145026 | c.98C>T | p.T33I | Heterozygous | 0/400 | No |
|  |  |  | c.1973T>C | p.V658A | Heterozygous | 0/400 | No |
| **NT-8-1** | *WFS1* | NM_006005 | c.623A>G | p.N208S | Heterozygous | 0/400 | No |
| **NT-9-1** | *DIAPH1* | NM_005219 | c.2156C>A | p.P719H | Heterozygous | 0/400 | No |
|  | *MYH9* | NM_002473 | c.2714G>A | p.R905H | Heterozygous | 0/400 | No |
|  | *TECTA* | NM_005422 | c.3605C>T | p.S1202F | Heterozygous | 0/400 | No |
| **NT-10-1** | *SEMA3E* | NM_012431 | c.1732G>A | p.V578I | Heterozygous | 0/400 | No |
