# Supplementary figures and images for: Genetic Etiology Study of Ten Chinese Families with Nonsyndromic Hearing Loss
Source: Neural Plast. 2018 Jul 5;2018:4920980. doi: 10.1155/2018/4920980 (PMC6079373; doi:10.1155/2018/4920980)

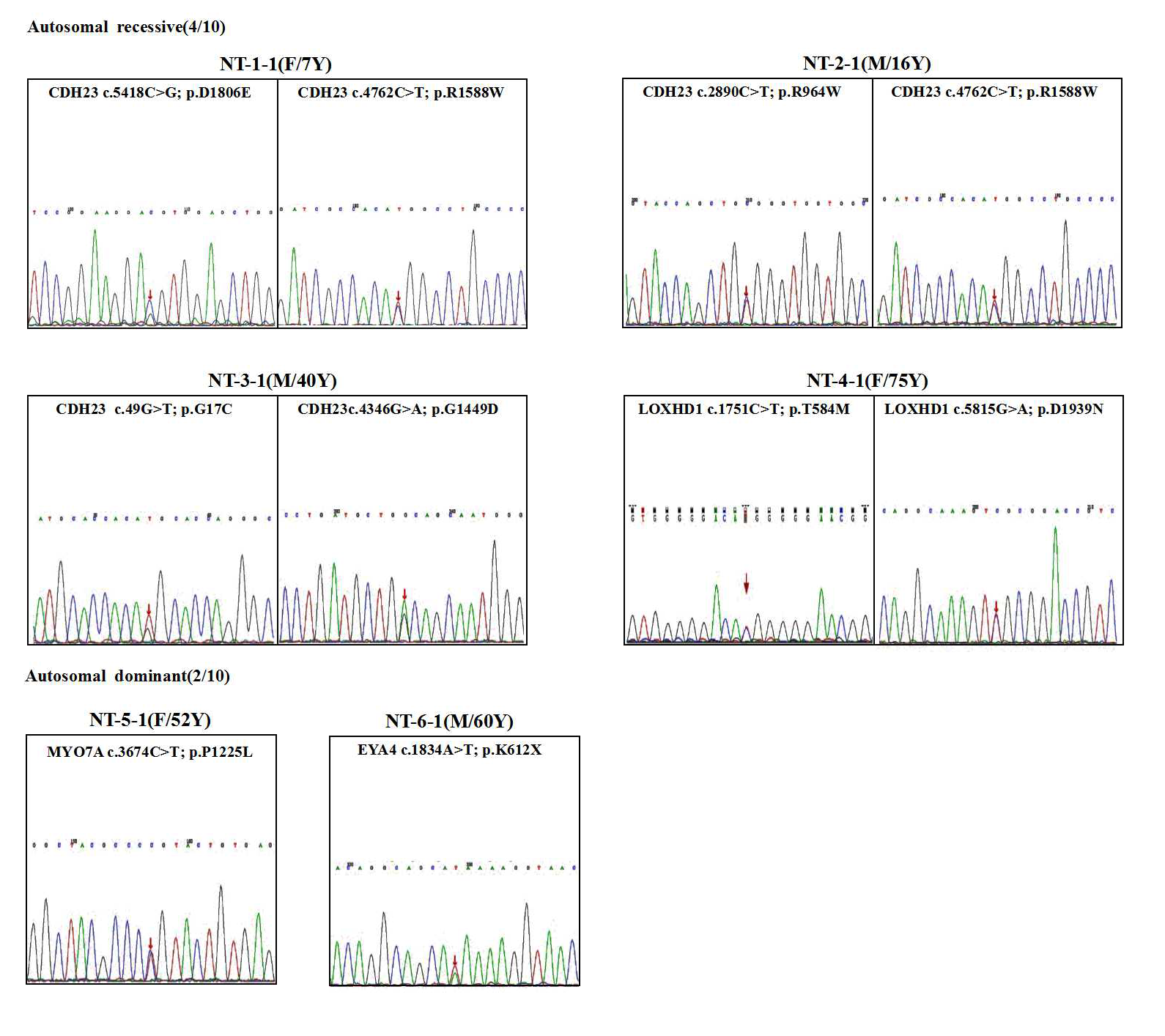

Supplement: Supplementary 3 — Figure 1: validation of candidate mutations by PCR-Sanger sequencing. [file 4920980.f3.tif]
