# Supplementary material for: Genetic Etiology Study of Ten Chinese Families with Nonsyndromic Hearing Loss
Source: Neural Plast. 2018 Jul 5;2018:4920980. doi: 10.1155/2018/4920980 (PMC6079373; doi:10.1155/2018/4920980)
Supplement: Supplementary 4 — Function analysis on candidate mutations. [file 4920980.f4.docx]

**Supplementary material file 4:** Function analysis on candidate mutations.

Evolutionary conservation and structure of wild-type and mutant-type created by SWISS-MODEL:

b

***CDH23***：


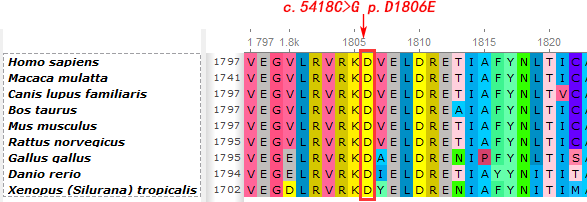


a


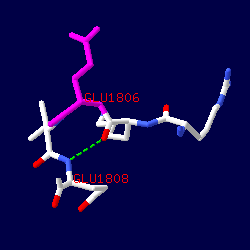

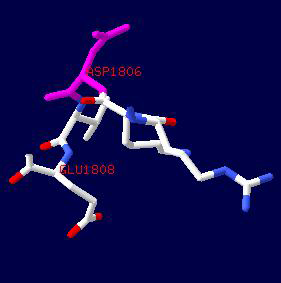


b

Wild（a），Mutation D1806E（b）: A hydrogen bond was formed between Glu of 1806 and Glu of 1808 after the mutation


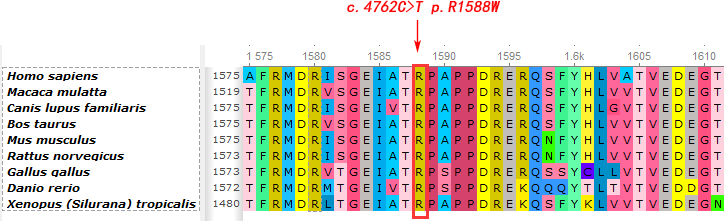


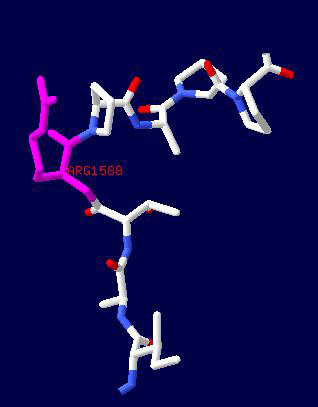

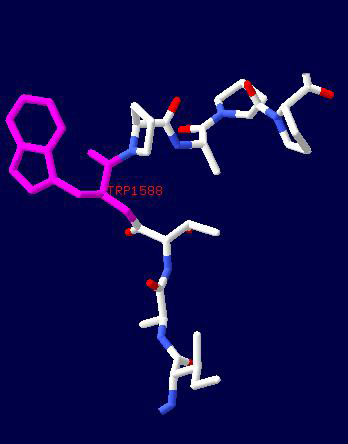


b

a

Wild（a），Mutation R1588W（b）: The side chain of the amino acids changed after the mutation


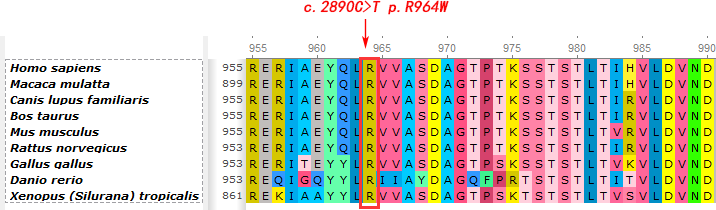


There is no protein structural homology-model for p.R964W.

There is no protein structural homology-model for p.G17C .


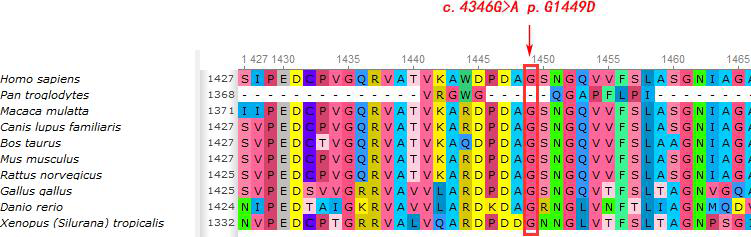


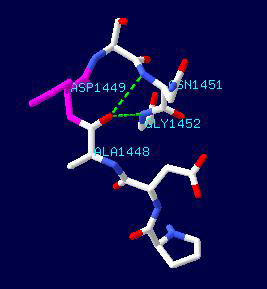

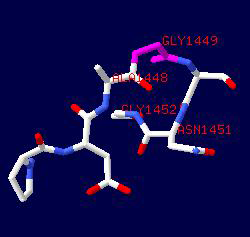


a

b

b

Wild（a），Mutation G1449D（b）: A hydrogen bond was formed between 1448 alanine (Ala) and 1,451 asparagine (Asn) and 1,452 glycine (Gly) after the 1449 glycine (Gly) mutation.

***LOXHD1***:


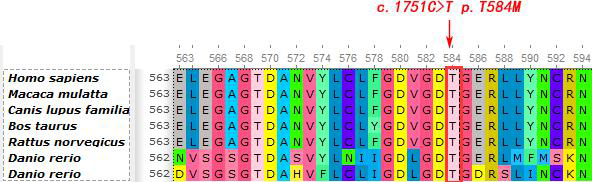


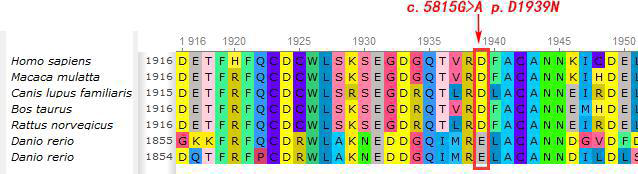


There is no protein structural homology-model for p.T584M and p.D1939N.

***MYO7A***:


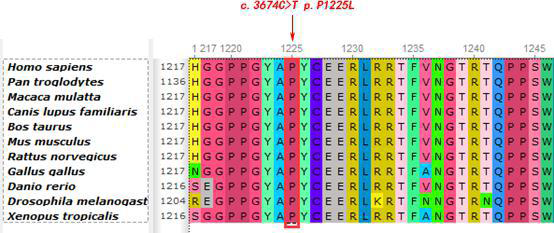


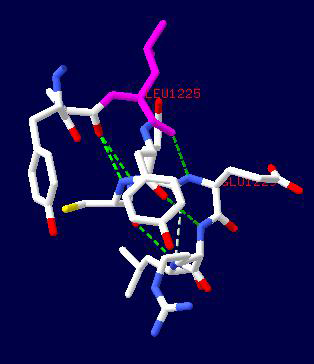

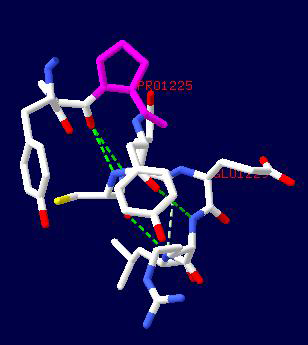


b

a

Wild（a），Mutation P1225L（b）: The side chain of the amino acids changed after the mutation.

***EYA4***:

a

c

b


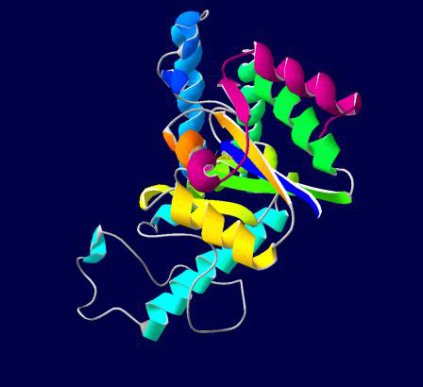

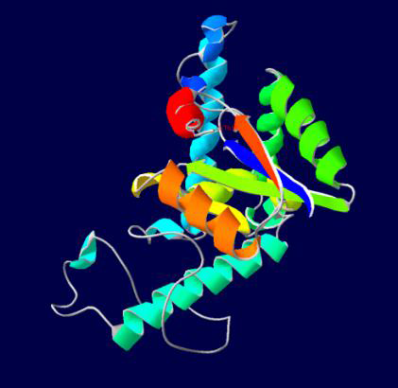

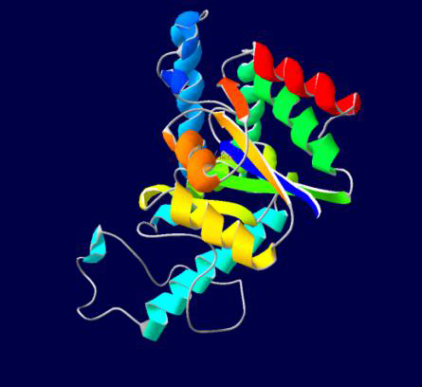


Wild（a），Mutation K612X（b），The missing parts are highlighted in magenta（c）
